# Physicochemical Analysis and Digestive Enzymes Inhibition of a Selected Malaysian *Apis cerana* Honey

**DOI:** 10.3390/foods14223958

**Published:** 2025-11-19

**Authors:** Suraiami Mustar, Nurliayana Ibrahim, Noor Athirah Pauzi, Aswir Abd Rashed, Mohd Fairulnizal Md Noh

**Affiliations:** 1 Nutrition Unit, Nutrition, Metabolism and Cardiovascular Research Centre, Institute for Medical Research, National Institutes of Health, Ministry of Health Malaysia, Block C7, Level 3, No. 1, Jalan Setia Murni U13/52, Seksyen U13, Setia Alam 40170, Selangor, Malaysia; liayana.psh@moh.gov.my (N.I.); noorathirah.psh@moh.gov.my (N.A.P.); aswir@moh.gov.my (A.A.R.); 2 Nutrition, Metabolism and Cardiovascular Research Centre, Institute for Medical Research, National Institutes of Health, Ministry of Health Malaysia, Block C6, Level 3, No. 1, Jalan Setia Murni U13/52, Seksyen U13, Setia Alam 40170, Selangor, Malaysia; fairulnizal@moh.gov.my

**Keywords:** *Apis cerana*, Malaysian honey, physicochemical, pancreatic lipase, α-amylase, α-glucosidase, anti-obesity, anti-diabetic

## Abstract

The Malaysian *Apis cerana* honey (ACH) was analysed for its physicochemical characteristics, including moisture, Baume, Brix analysis (total soluble solids and total soluble sugars), sugar profiling (fructose, glucose, sucrose, maltose, and lactose), total ash, pH, free acidity, electrical conductivity, colour analysis, and choline content. The inhibitory effects of pancreatic lipase, α-amylase, and α-glucosidase activities were also assessed. Results indicated that the sum of fructose and glucose, sucrose, and electrical conductivity were all within the recommended range following the requirements of international standards. The maximum levels were, nevertheless, exceeded by moisture, free acidity and total ash. The ACH showed potential as an anti-obesity and anti-diabetic agent by inhibiting pancreatic lipase by up to 43.4% at 0.063 mg/mL, α-amylase by up to 70% at 7.0 mg/mL and α-glucosidase by up to 67.6% at 100 mg/mL, respectively. The percentage inhibition of α-glucosidase by undiluted ACH and deionised water extract at different temperatures (4.8 ± 0.5 °C, 27 ± 0.5 °C, and 40 ± 0.5 °C) was comparable, suggesting that temperatures had little effect on the degree of inhibition.

## 1. Introduction

Obesity and diabetes are the two prominent non-communicable diseases that have a significant impact on the world’s population today [1,2]. Many ways have been suggested and used to overcome both diseases that cause a burden to humans and nations. The implications of obesity and diabetes are causing more health problems, leading to other ailments such as heart disease and kidney problems, reduced productivity, quality of life, and longevity [3]. Several medications found on the market to treat obese patients include orlistat, liraglutide, and naltrexone/bupropion. All these medications use different pathways to defeat obesity [4]. For diabetes, drugs commonly used include metformin, acarbose, repaglinide, and pioglitazone, which are prescribed according to the needs of the diabetic patients [5].

One possible way to overcome obesity and diabetes is by inhibiting the digestive enzymes involved in their mechanisms. Inhibiting pancreatic lipase, associated with fat uptake in the small intestinal tract, is one way to treat obesity [6]. Among the three drugs for obesity treatment, only orlistat acts by inhibiting pancreatic lipase. However, orlistat has several side effects, such as causing diarrhoea, oily stools, and the inability to control urination, which limits its usage [4]. Diabetes treatment comprises inhibiting the digestive enzymes α-amylase and α-glucosidase involved in glucose uptake in the small intestine [7]. Acarbose, one of the medications used to treat diabetic patients, functions by inhibiting both α-amylase and α-glucosidase. Even then, utilising acarbose has adverse effects that might cause discomfort to the patients, involving itchiness, moderate diarrhoea, bloating, and stomach pain [8]. Therefore, it is necessary to find other alternative solutions to address the problems of using natural products that might have fewer side effects and replace the use of synthetic drugs available on the market.

Functional foods and naturally occurring bioactive compounds derived from plants have shown potential in regulating essential metabolic pathways involved in fat and glucose metabolism, with fewer side effects. Among these natural functional foods, honey has attracted significant attention due to its unique complex mixture of sugars, phenolic compounds, vitamins, and minerals [9]. Several studies indicate that honey from different floral sources and bee species (both stinging and stingless) can inhibit α-amylase and α-glucosidase [10,11], while reports on pancreatic lipase inhibition remain limited [12]. These beneficial effects are mainly attributed to its bioactive compounds, especially polyphenols [13,14]. The differences in enzyme-inhibitory activity among honey types suggest that botanical and entomological factors significantly influence their bioactivity [15].

In Malaysia, the most common honey available in the market and mostly produced by local bee farmers from the Apis species is *Apis mellifera* honey. Conversely, *Apis cerana* honey (ACH) is relatively underexplored due to limited resources [16]. Bee farmers often face challenges in raising this species due to their cautious or delicate nature [17]. *A. cerana*, which is also smaller than *A. mellifera*, produces less honey, affecting the farmers’ economy [17]. However, since the *Apis cerana* honeybee is native to Malaysia and widely distributed across Southeast Asia and other Asian regions [18], prioritising research on it should be the main focus. Being a local species, *Apis cerana* differs from the imported *A. mellifera*, which comes from temperate countries, and may have a distinct and unique phytochemical profile in its honey. This profile could confer different biological activities, including potential inhibitory effects on key digestive enzymes linked to metabolic diseases [19]. *A. cerana* bees gather nectar from vegetation, excretions from various parts of living plants, and secretions from insects living on different sections of plants. They modify these by combining them with their own components, evaporating the moisture, and storing them in the honeycomb for full development [20]. In this study, the plants and trees surrounding the *A. cerana* hives include Coral Vine, Power Puff, acacia, and coconut trees. This honey, which is light golden in colour, gradually darkens and has a pleasant aroma. However, there is currently a lack of scientific data regarding the functional potential and enzymatic inhibitory effects of ACH in Malaysia.

Therefore, this preliminary study aims to evaluate the physical and chemical characteristics (physicochemical) of ACH and its inhibitory effects on digestive enzymes related to obesity (pancreatic lipase) and diabetes (α-amylase and α-glucosidase), as well as to investigate the effect of temperature on enzyme inhibition.

## 2. Materials and Methods

### 2.1. Sample Collection

The ACH was obtained from Pusat Apiari Nasional, Parit Botak, Batu Pahat, Johor Darul Takzim, Malaysia. Several fresh and mature honeycombs were collected randomly from different bee hives of the same batch and filtered. The fresh sample was kept in a cooler box with coolants to maintain a low temperature (4 °C) until it reached the Institute for Medical Research (IMR) Laboratory, National Institutes of Health (NIH), at Setia Alam, Selangor, Malaysia. Then, it was kept in a freezer (−20 °C) until further analysis. The analysis was performed within one to six months of collection.

### 2.2. Physicochemical Analysis

The honey sample was analysed for moisture, Baume, Brix, sugar profiling (fructose, glucose, sucrose, maltose, and lactose), total ash, pH, free acidity, electrical conductivity, colour, and choline content. All experiments were conducted in triplicate. All these analyses were conducted to certify honey’s quality, authenticity, safety and its botanical origin based on the Malaysian Food Act (MFA) and Codex Alimentarius (CA), wherever applicable.

#### 2.2.1. Moisture

Moisture content was determined using three different methods: refractometry, following the International Honey Commission (IHC) [21], air-oven drying, following the AOAC (930.15) method [22], and a moisture analyser, following a standard procedure by Nielsen, 1994 [23]. A portable honey refractometer BOE 30106 (Boeco, Hamburg, Germany) with a scale of 12–27% for water was calibrated using a standard solution provided by the manufacturer before use. The air-oven drying method was performed by heating the samples in an oven (UN 30, Memmert, Schwabach, Germany) at 105 °C until a constant weight was achieved. The moisture analyser (Mark 3, Sartorius, Gottingen, Germany) was utilised by weighing at least 5 g of the honey sample and heating it to 105 °C until all the moisture had evaporated and reached a constant weight.

#### 2.2.2. Baume (Density)

Honey density is determined using the same honey refractometer BOE 30106, which has a Baume scale of 38–43 °Be′.

#### 2.2.3. Brix Value

The Brix value or total soluble solids or sugars in honey was analysed using the same honey refractometer BOE 30106, which has a 58–90% Brix scale.

#### 2.2.4. Sugar Profiling

A high-performance liquid chromatography (HPLC) Binary pump 2515 (Waters Corporation, Milford, MA, USA) equipped with a detector (refractive index) and column (XBridge Amide, 3.5 μm, size 4.6 × 250 mm), a validated in-house method was used for sugar profiling. The mobile phase mixture comprised 75:25 (*v*/*v*) acetonitrile and ultra-pure water with 0.2% triethylamine as the buffer. One mL per minute of flow rate was retained when a 20 μL sample was administered into the HPLC. Throughout the 15 min runtime analysis, the column temperature was kept at 55 °C. Mixed standard solutions of fructose, glucose, lactose, maltose, and sucrose at 1% of each solution were prepared in 50% acetonitrile/water. Working sugar mixture solutions of 0.1%, 0.2%, 0.5%, and 0.8% were prepared from the 1% mixed standard solution. In a capped 50 mL conical tube, ACH at 1 g was extensively homogenised in 25 mL of acetonitrile/water. Later, the resulting mixture underwent filtering using a 0.45 μm nylon filter. Before sample analysis, the quality control sample (cordial) was analysed simultaneously after standard calibration to ensure the reliability of the data. The control was accepted if it fell within two standard deviations (2SD) of the mean value, previously set as the acceptable limit for the control.

#### 2.2.5. pH and Free Acidity

The pH level was recorded using a pH electrode (Isolab, Eschau, Germany). Honey’s free acidity content was quantified using the titration method. Diluted honey at 10 g in 75 mL of carbon dioxide-free water was titrated with sodium hydroxide (NaOH, 0.1 M) until the pH reached 8.30. The titrant volume was expressed to the closest 0.2 mL, using a 10 mL burette [21]. The free acidity was expressed as milliequivalents acid/1000 g honey (meq/1000 g).

Free acidity (meq/1000 g) = mL of 0.1 M NaOH × 10

#### 2.2.6. Ash

The ACH was heated at 600 °C in a muffle furnace (Carbolite Gero, Neuhausen, Germany) until it reached a consistent weight following the International Honey Commission’s (IHC) technique [11]. Five grams of the sample in a quartz dish were weighed to the closest 0.001 g. Pre-evaporation and preliminary ashing were performed on a hotplate. Later, the dish was ashes for 2 h in a furnace and chilled in a desiccator. The procedure was repeated until a steady weight was achieved [21].

#### 2.2.7. Electrical Conductivity

The IHC procedure [21] was adapted to ascertain the electrical conductivity of honey’s solution dry matter at 20% (*w*/*v*) using an electrical conductivity metre (Isolab, Germany). Cell constant was determined using a potassium chloride solution and computed by employing the following equation:

(1) $$K=\frac{11.691}{G}$$

In which;

- K = cell constant (cm^−1^)
- G = electrical conductance (mS), as obtained using a conductivity cell
- 11.691 = electrical conductivity of fresh distilled water (mS/cm) and 0.1 M potassium chloride solution, total value means at 20 °C

Following the cell constant confirmation, honey’s electrical conductivity was calculated as below:

(2) S_H_ = K × G

In which;

- S_H_ = honey’s electrical conductivity (mS/cm)
- K = cell constant (cm^−1^), calculated in Equation (1)
- G = conductance (mS)

The final electrical conductivity value is presented with a 0.01 precision (mS/cm)

#### 2.2.8. Colour Analysis

A colour photometer (Hanna Instruments, Woonsocket, RI, USA) based on the Pfund classifier was utilised to evaluate the honey’s colour. Four mL of a homogenous sample, without air bubbles, was inserted into the cuvette-based photometer. In comparison to the glycerol reference analytical quality, results were presented in millimetre Pfund (mm) grades as specified by the United States Department of Agriculture (USDA) certified colour quality measurement [24]. Honey colour ranges from water white (0–8 mm), extra white (>8–17 mm), white (>17–34 mm), extra light amber (>34–50 mm), light amber (>50–85 mm), amber (>85–114 mm) and dark amber (>114 m).

#### 2.2.9. Choline

Choline content was analysed with an ICS-2100 ion chromatography system (ICS) (Thermo Fischer Scientific, Waltham, MA, USA) fitted with a suppressed conductivity detector. Analysis was performed using 5.0 mM methanesulfonic acid as the mobile phase and an analysis time of 17 min. One gram of the honey sample or control standard (infant milk powder) in a test tube was digested with 2.5 mL hydrochloric acid (1 M) and kept warm at 70 °C for 3 h. Later, it was chilled at ambient conditions, mixed with 21.5 mL of ultra-pure water, and filtered using ashless filter paper after centrifuging at 4000 rpm for 12 min. The solution, when kept at 4 °C, was stable for up to 3 days. The choline analysis required ten millilitres of the solution to be introduced into the IC system, with an average flow of 1.0 mL/min. After standard calibration, the control (infant milk powder) was analysed, and the results must be within 2SD before analysing the sample to ensure data reliability.

### 2.3. Enzyme Analysis

The inhibition of digestive enzyme activities (pancreatic lipase, α-amylase, and α-glucosidase) was determined using standard methods or established techniques.

#### 2.3.1. Inhibition of the Pancreatic Lipase Activity

A turbidimetric technique, slightly modified from the approach by Chater et al. (2016) [25], was employed to determine pancreatic lipase activity. A buffer diluent (0.033 M citric acid, 0.343 M potassium hydroxide, 0.033 M orthophosphoric acid, 0.033 M boric acid, and 0.35% taurodeoxycholate sodium salt) with a pH of 7.3 was prepared. Then, 4 mL of free fatty acid olive oil solution (1% olive oil in acetone) was added to 100 mL of hot diluent buffer, sustained at 70 °C on a hot plate, and homogenised at high speed for 10 min to prepare the substrate solution. For enzyme preparation, 1 mg of lipase (0.5 U/mL) was diluted in 1 mL of buffer diluent. The sample (0.004–0.5 mg/mL) was prepared by homogenising honey with the prepared substrate solution for 2 min at room temperature (25 °C). A similar process was used to prepare orlistat (at 0.063 mg/mL), a positive control for inhibition. Two 96-well microplates were used for each experiment, with plate 1 containing 10 µL of lipase enzyme or buffer diluent and plate 2 containing 240 µL of orlistat and the sample solution at various concentrations. All assays were run three times. Absorbance at 405 nm was taken at every 5 min intervals using a spectrophotometer. The pancreatic lipase was used as the control representing 100% activity. The differences in absorbance reading from time point zero were calculated for each substrate solution:

∆Absorbance = Absorbance *T*_0_ − Absorbance *T_x_*

Lipase activity (%): % activity relative to the substrate control at

$$T_{x}=\frac{\Delta \mathrm{Absorbance}\;\mathrm{sample}}{\Delta \mathrm{Absorbance}\;\mathrm{control}}$$

% inhibition = 100% − lipase activity (%)

#### 2.3.2. Inhibition of the α-Amylase Activity

Determination of α-amylase activity was performed following a modified method by Balasubramaniam et al. (2013) [26]. Sorenson Phosphate buffer (SPB) was prepared by mixing 9.08 g potassium dihydrogen phosphate (KH_2_PO_4_) and 11.88 g disodium hydrogen phosphate dihydrate (Na_2_HPO_4_·2H_2_O) in 1 L of distilled water and pH adjusted to 7.0. Honey samples (0.8 mg/mL), α-amylase enzyme (0.25 mg/mL), positive control (acarbose, 3 mg/mL), and maltose standards (0–20 mg/mL) were diluted with the SPB. A 96-well plate was used for the assay. Acarbose and honey samples at different concentrations, with α-amylase, were incubated for 10 min at 37 °C. A starch solution at 0.5% in SPB was heated until solubilised, and cooled at ambient conditions. Later, it was added to each well at 60 µL. The mixture was retained for half an hour at 37 °C. Subsequently, 120 µL of DNS reagent was added and maintained at 100 °C for 15 min. The plate was cooled at ambient conditions, and absorbance at 550 nm was determined.

$$\%\;\mathrm{inhibition}=\frac{\left(\Delta Abs_{control}-\Delta Abs_{sample}\right)}{\Delta Abs_{control}}\times 100$$

#### 2.3.3. Inhibition of the α-Glucosidase Activity

A colourimetric method adapted from Apostolidis et al. (2011) [27] was employed to evaluate the α-glucosidase activity at 405 nm. A chemical compound, 4-nitrophenyl β-D-glucopyranoside (PNPG), was used as the substrate. A 96-well microplate was filled with 50 μL of honey samples/acarbose (1.5 µM)/0.1 M phosphate buffer, pH 6.9, and 1.0 U/mL of α-glucosidase solution (100 μL) and retained at room temperature for 10 min. The absorbance reading at 405 nm was obtained after pre-incubation, adding 50 μL of 5 mM PNPG, and a 5 min incubation at room temperature. The absorbance was once more determined, and the assay was conducted in triplicate.

$$\%\;\mathrm{inhibition}=\frac{\left(\Delta Abs_{control}-\Delta Abs_{sample}\right)}{\Delta Abs_{control}}\times 100$$

#### 2.3.4. Different Treatments of Honey on α-Glucosidase Activity

The ACH at 100 mg/mL was either undiluted or diluted with deionised water at different temperatures: 4.8 ± 0.5 °C (cold), 27 ± 0.5 °C (room), and 40 ± 0.5 °C (warm). These specific temperatures were chosen to represent normal honey usage, where honey is consumed in its usual undiluted form or diluted in cold, room, or warm water. The honey solutions were vortexed for 10 min and later dried using nitrogen gas (to maintain nutritional and bioactive properties). The remaining dried solutions (deionised water extract) were then subjected to the inhibition of α-glucosidase activity as mentioned in Section 2.3.3.

### 2.4. Statistical Analyses

All analyses or experiments were performed three times. GraphPad Prism Software (version 5.0) was used to determine IC_50_ values, descriptive statistics, and one-way ANOVA analysis employing the Tukey post hoc test. A statistically significant *p*-value was set at *p* < 0.05.

### 2.5. Flowchart of the Study

**Figure 1:**
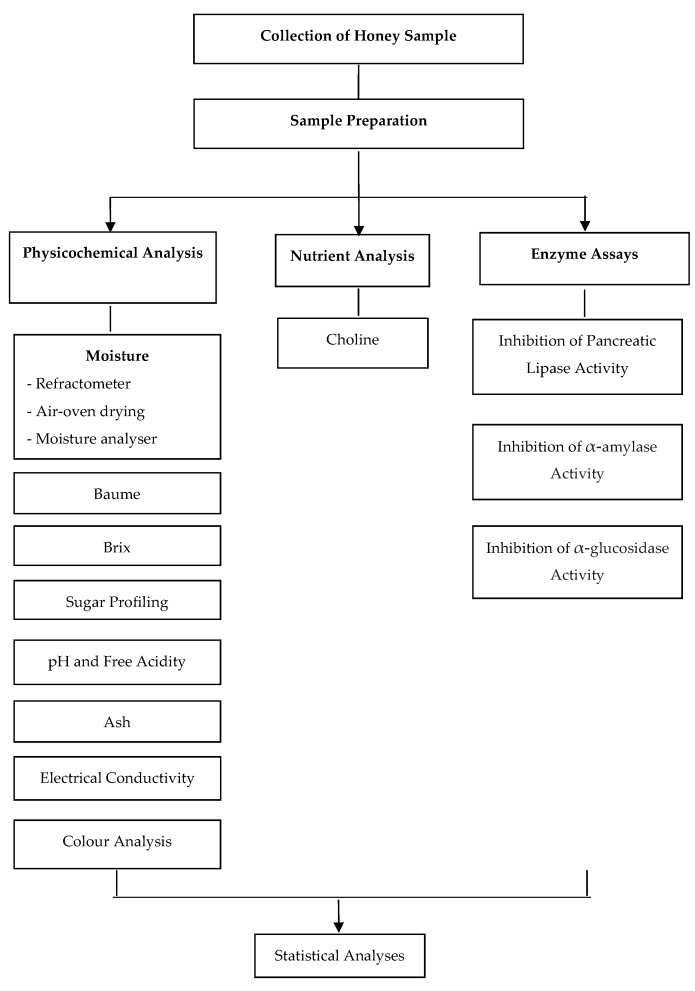
Schematic diagram of the study.

Figure 1 illustrates the study flow from sample collection to laboratory analysis and data analysis using statistical software.

## 3. Results

### 3.1. Physicochemical Analysis

Among the parameters with legislative allowable limits are moisture content (using a refractometer), the sum of fructose (F) and glucose (G), sucrose, free acidity, total ash, and electrical conductivity. Overall results indicated several parameters exceeded standard limits, including moisture, free acidity, and ash, while sugar profiles remained within acceptable ranges. Table 1 presents the detailed physicochemical analysis of *Apis cerana* honey (ACH) compared with the national and international standards.

Moisture content was determined using three different methods, and the lowest reading was observed with the moisture analyser, at 8.6 ± 0.5%, followed by the refractometer (22.7 ± 0.1%) and finally by air-oven drying (24.4 ± 0.2%). The moisture content of the ACH was 2.7% higher (refractometer) than the standard value for all honey types (<20%), but following the Heather honey (Hea) (<23%), indicating a higher moisture level in comparison to other *A. mellifera* honey.

A refractometer has been designed to enable the determination of honey moisture/Baume/Brix readings at the same time. The ACH had an estimated density (Baume) of 40.1 ± 0.0 °Be′ and a Brix value of 75.5 ± 0.1%. Codex Alimentarius (CA) [20] and International Honey Commission (IHC) [21] have not included both tests for honey standards, and the readings of Baume and Brix are additional information on honey characteristics associated with its density and total soluble solids or sugars.

Individual sugar was analysed using HPLC, and the results in Table 1 show that the content of fructose (F) was 29.75 ± 0.27 g/100 g, glucose (G) 28.76 ± 0.23 g/100 g, and the sum of F and G was 58.81 ± 0.50 g/100 g, following the limit set by the international standard of at least 45% for honeydew honey. The sucrose content was 8.44 ± 0.06 g/100 g following the standard value of <10% set by the MFA for all types of honey and CA of <10 g/100 g for honey, among others, such as Alfalfa, Citrus spp., Red Gum, Leatherwood, and False Acacia. The reading for maltose was quite low, at 0.59 ± 0.00 g/100 g, and negligible for lactose.

Honey is naturally acidic and has a wide pH range, from the lowest at 3.1 to the highest at 7.05 [29,30]. The ACH had a pH of 4.13 ± 0.01 (Table 1), within the range reported by other researchers from earlier studies [31,32,33]. For free acidity, the CA [20] has proposed a higher maximum value of less than 50 meq/1000 g, in contrast to the MFA [28], which recommended a value of less than 40 meq/1000 g for acidity in honey. Compared to the suggested standard values, the free acidity of ACH was 93.7 ± 0.6 meq/1000 g, which is considered high.

The limit value for ash content in honey, as proposed by national and international standards, is less than 1.0 g/100 g or 1%. The ACH sample tested indicated a higher ash content of 2.8 ± 0.5%.

ACH had an electrical conductivity of 1.66 ± 0.00 mS/cm, greater than 0.8 mS/cm suggested for honeydew and chestnut honey, or a combination of the two. The elevated conductivity strongly suggests that ACH appeared to be more characteristic of honeydew-type honey, rather than floral nectar [20]. However, further investigations are needed to confirm this observation.

Regarding the colour of honey, it was designated using the USDA Colour Standards and is classified into seven classes, ranging from water-white to dark amber. The Pfund colour scale (mm) range for honey is <9 to >114 mm. The ACH was in the dark amber range (150.0 ± 0.0 mm Pfund), being in the darkest colour range available for honey.

Apart from the physical and chemical characteristics of ACH, we also investigated the presence of choline in the sample since it is one of the essential nutrients required for optimal health. Unfortunately, choline was not detected in the ACH sample using the ICS.

### 3.2. Enzyme Analysis

#### 3.2.1. Pancreatic Lipase Activity

**Figure 2:**
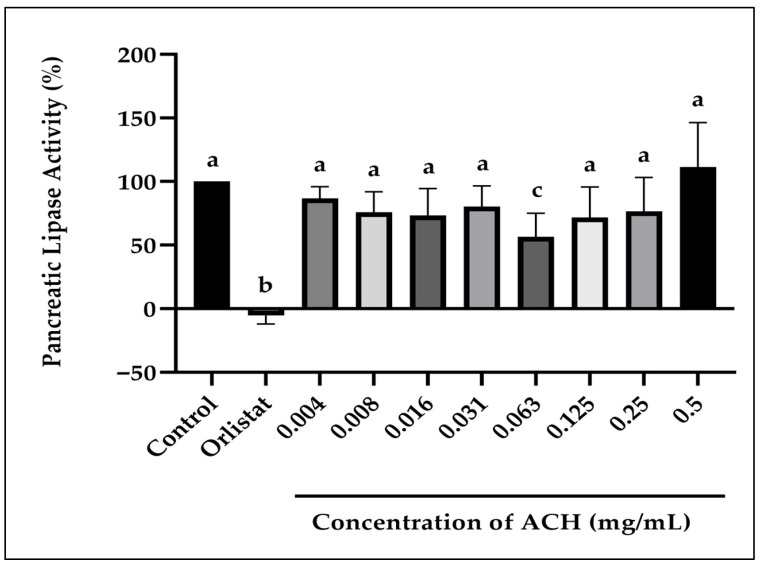
The inhibition of pancreatic lipase activity by ACH. Positive control (Orlistat) is at 0.063 mg/mL. Different letters (a, b, and c) denote statistically significant differences at *p* < 0.05.

The percentage of inhibition of pancreatic lipase activity of ACH ranged from 13.2 to 43.4% at concentrations of 0.004 to 0.25 mg/mL (Figure 2). The inhibition was not dose-dependent. These results exhibited a peak inhibition effect at 0.063 mg/mL, statistically significant (*p* = 0.006) compared to the control. At concentration levels lower and higher than 0.063 mg/mL, the efficacy was reduced. The positive control (orlistat) at 0.063 mg/mL is potent against lipase, significantly inhibiting it by more than 100%.

#### 3.2.2. α-Amylase Activity

**Figure 3:**
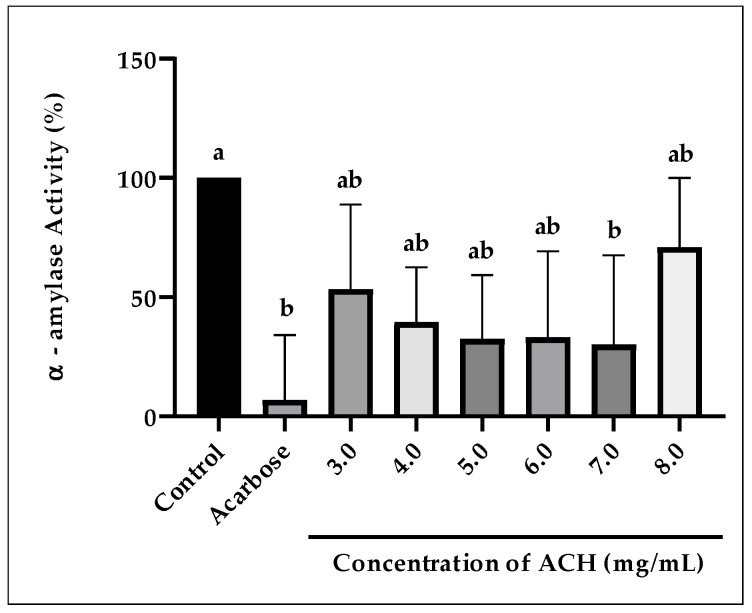
The inhibition of α-amylase activity by ACH. Positive control (Acarbose) is at 3.0 mg/mL. Different letters (a and b) denote statistically significant differences at *p* < 0.05.

Results in Figure 3 feature the inhibition of ACH on α-amylase activity in the range of 3.0 to 8.0 mg/mL. The inhibition was not in a dose–response manner (non-linear or irregular), with the percentage of inhibition for each concentration tested being 46.7, 60.6, 67.6, 66.9, 70.0, and 29.1%, respectively. The highest inhibition was at 7.0 mg/mL, with a percentage of inhibition 70%, and was statistically significant (*p* = 0.042) compared to the control, but not to acarbose (*p* = 0.944). At other concentrations, the inhibition was not statistically significant (*p* > 0.05), either compared to the control or acarbose, at a percentage of inhibition ranging from 46.7 to 66.9%. A positive control, acarbose, at 3.0 mg/mL showed the highest inhibition of 93.2%, demonstrating potent activity against α-amylase.

#### 3.2.3. α-Glucosidase Activity

**Figure 4:**
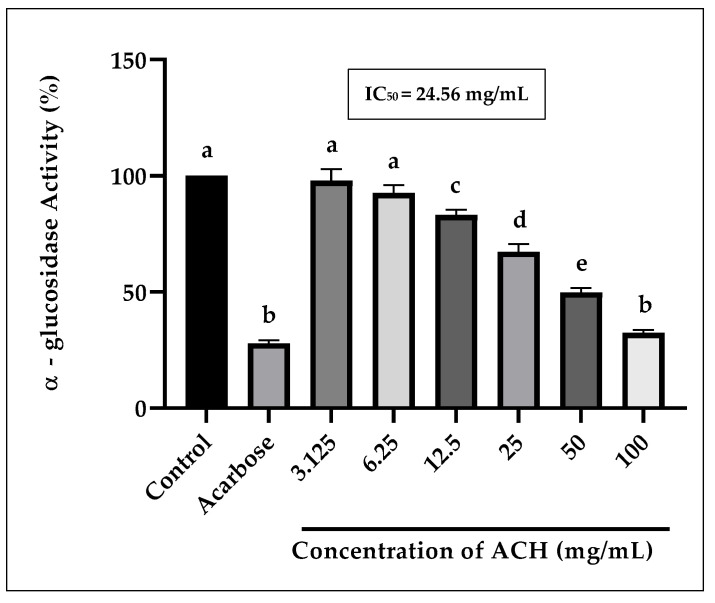
The inhibition of α-glucosidase activity by ACH. Positive control (Acarbose) is at 4.0 mg/mL. Different letters (a, b, c, d, and e) denote statistically significant differences at *p* < 0.05.

Figure 4 highlights the inhibition of ACH on α-glucosidase activity, with a percentage of inhibition ranging between 2.1 and 67.6% for concentrations of 3.125 to 100 mg/mL. According to the results, elevated concentrations of ACH were found to enhance inhibitions. The inhibition was in a dose–response manner, with a calculated IC_50_ value of 24.56 mg/mL, higher than the positive control, acarbose (2.04 mg/mL), showing the least potency towards α-glucosidase, as compared to acarbose.

**Figure 5:**
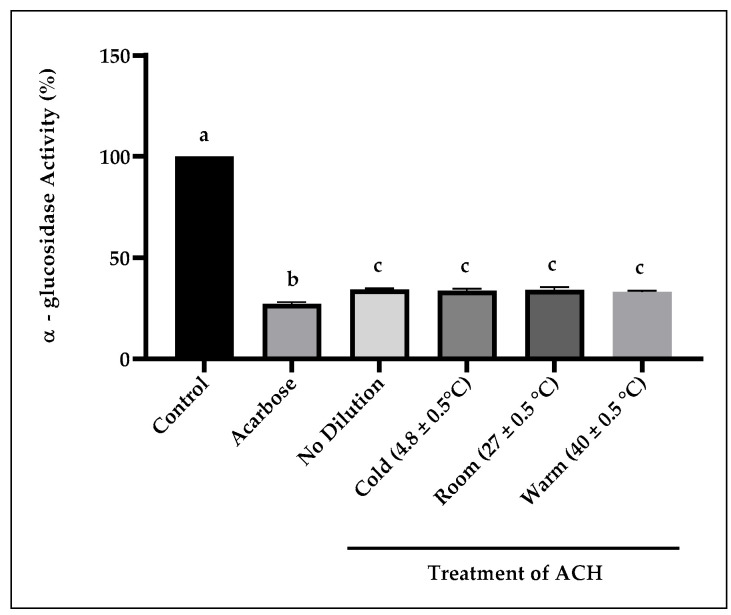
The inhibition of α-glucosidase activity by ACH at different temperatures of diluting solution treatments. Positive control (Acarbose) is at 4.0 mg/mL. Different letters (a, b, and c) denote statistically significant differences at *p* < 0.0001.

Figure 5 presents the α-glucosidase inhibition of undiluted and diluted ACH (100 mg/mL) following treatment at different temperatures (cold: 4.8, room: 27.0, and warm: 40 °C). Results show that the inhibition between undiluted and all diluted ACH in deionised water (extracts) was not significantly different, with percentages of inhibition at 65.7, 66.3, 65.9, and 66.9%, respectively. But when compared to the control, they were statistically significant at *p* < 0.0001. Acarbose was more potent, with an inhibition of 72.9% at 4.0 mg/mL, and when compared to ACH and the control, it was significantly different at *p* < 0.0001.

## 4. Discussion

### 4.1. Physicochemical Analysis

Various methods used to determine moisture content produced vastly different results, indicating that methods influence moisture content outcomes. Compared to the other two methods, the values obtained with the moisture analyser were substantially lower. This discrepancy may be attributed to the divergent principles used for each piece of equipment and methodological factors. Moisture analysers depend on rapid thermal drying; high temperature settings or extensive heating may result in excessive drying or destruction of volatile compounds, leading to inaccurately low readings. Inadequate instrument calibration or uneven sample distribution can also contribute to error [34]. In contrast, refractometry is a standardised method for moisture analysis in honey according to the Codex Alimentarius (CA) guidelines [20] and the International Honey Commission (IHC) [21], due to its rapid, standardised, consistent, non-destructive, and reliable estimates of true moisture content.

CA [20] proposed a moisture value of less than 23% for Heather honey (Hea) and less than 20% for other varieties. The proposed values were primarily derived from honey manufactured by the *A. mellifera* honeybee from temperate countries. The MFA has adopted the same value without adjustment for the tropical climate [28]. The moisture content of ACH exceeded both guidelines and raises critical questions about the authenticity, maturity, and applicability of these universal standards for honey. The non-compliance, however, may be due to natural variations rather than poor quality. Honey absorbs more moisture because of Malaysia’s tropical humidity, and *A. cerana* bees may naturally produce honey with higher moisture content than *A. mellifera* bees, for which these guidelines were created. Increased moisture could result from postharvest procedures, including processing and storage [35], which theoretically heightens the risk of fermentation [36]. Nonetheless, research indicates that there was no correlation between higher moisture and spoilage [37]. These findings highlight the need for a different or specific quality standard, designed for tropical *A. cerana* honey, rather than framing it as inferior or less valuable. Standards should reflect true biological differences, rather than inappropriately applying temperate-climate norms to a substantially different product.

The Baume value (°Be′) estimates the density of honey, and the percentage of total soluble solids or sugars is reflected by the Brix value [38]. However, studies on the Baume values in honey were scarce. A pycnometer is more often used to determine the density of honey rather than a refractometer, which is often believed to be more accurate but labour-intensive and time-consuming [39,40].

The Brix values for other types of honey, such as Acacia (78.08–82.27%), Linden (79.20–83.26%), and multifloral (77.83–82.67%), as reported in a previous study [41], show that honey has a Brix value between 75% and 85%. The ACH was within that range with an acquired value of 75.5%.

Honey crystallisation is affected by several parameters: fructose-to-glucose (F/G) ratio, sugar concentration, water-insoluble substances, temperature, and storage conditions [42]. The honey will be more susceptible to crystallisation if the proportion of F/G is lower than 1.0 [42]. The ratio of F/G for ACH was 1.03, indicating less susceptibility to form a crystal, making it easier to handle and ideal for commercial food products. The sucrose content of ACH was <10% or <10 g/100 g. These readings, which were within the accepted level for national and international standards, suggested that the ACH sample might not be adulterated with white sugars, which can offer significant health benefits. No standard values, however, have been proposed for both maltose and lactose content in honey.

Although pH is not included as a honey standard, pH value is one of the important factors in controlling food spoilage, since low pH discourages the proliferation of microorganisms and maintains stability during storage [43]. According to several studies, honey’s pH may vary between 3.26 and 4.01 [43], from 3.67 to 4.11 [44], and from 3.3 to 5.3 [45], regardless of their geographical origin. The ACH had an acidic pH of 4.13, within the range of the previous studies. The acidity helps honey to maintain its shelf life by inhibiting the growth of many microorganisms [43].

The free acidity of ACH was more than double the MFA maximum level, which significantly exceeds the regulatory limit. Although enhanced free acidity is frequently linked with fermentation or degradation [46,47], this interpretation might be overly simplistic for tropical *A. cerana* honey. Organic acids, particularly gluconic acid, synthesised during nectar development, are the primary source of free acidity [48]. Higher acidity may naturally come from tropical nectar sources and *A. cerana’s* unique glucose oxidase activity. High acidity may reflect botanical origin rather than quality imperfections [48], indicating that some floral sources naturally produce more acidic honey. More studies are required to differentiate between the consequences of actual deterioration (immature honey, poor storage, fermentation) and the natural variations in the ACH that might have been mistakenly defined or categorised by unsuitable standards.

ACH also exhibited elevated ash content, which raises concerns about its botanical origin and quality classification, because it surpasses the MFA [28] and Codex criteria [20]. Saudi Arabian honey has been shown to have similarly elevated ash levels (>1.0%) [49] and extreme cases (3.0–10.0%) [50], thus this disparity demands careful evaluation. A honeydew origin with a high mineral content (potassium, calcium, and magnesium) from plant exudates, rather than floral nectar, is typically identified by an ash concentration greater than 1% [51,52]. Nonetheless, there are several plausible explanations: *A. cerana’s* tropical foraging behaviours naturally produce mineral-rich floral honey [53], suggesting that temperate standards are inadequate; the honey is a mixed floral-honeydew origin or is contaminated with honeydew or other external contamination during harvesting, which artificially raises ash levels. Differentiating between these scenarios is challenging but can be overcome with melissopalynological analysis and mineral profiling [51,52].

The electrical conductivity of ACH was measured at 1.66 ± 0.00 mS/cm, which is notably higher than the 0.8 mS/cm threshold typically associated with honeydew and chestnut honeys [21]. ACH is seen as more inclined to honeydew-type owing to its high electrical conductivity value (increased levels of minerals and organic acids) [21], considering its high ash content and lower sum of fructose and glucose levels, which reflects the honeydew-type, originating from plant exudates or insect secretions rather than floral nectar [20]. However, this remains an evolving hypothesis rather than a confirmed classification, owing to several uncertainties. For instance, some floral honeys demonstrate higher values than honeydew honey, making electrical conductivity an unreliable sole indicator [20]. To accurately identify honeydew honey, comprehensive analyses such as melissopalynological examination to determine honeydew constituents and pollen spectrum [51], sensory assessments for unique flavour profiles, and chemical indicators including specific oligosaccharides, proline content, and particular rotation values [51] are essential. It is not possible to entirely exclude other potential causes, such as contamination, distinctive tropical floral sources, or nectar production specific to *A. cerana*. Further investigations combining these various analytical approaches are required to determine if ACH truly represents or resembles honeydew honey or reflects an independent phenomenon.

Differences in honey colour are due to the flora type, seasonal variation, extraction process (centrifugation, pressing, or draining), and processing parameters (exposure to heat, time, and storage conditions) [18,54]. The diverse pigments in various honey varieties also influence the varying colours [55]. Colour grading in honey is important because it reflects both consumer preference and botanical origin. Consumers often associate lighter honeys with a milder flavour and darker honeys, like the ACH, with a stronger taste and higher nutrient content [56]. As the Pfund value increases, the colour intensity and phenolic content typically rise, influencing nutritional quality and market value [57].

Humans require choline as a nutrient to maintain healthy well-being since it is a bioactive compound involved in several vital physiological processes [58]. Measuring choline in honey helps determine its nutritional and functional value, as certain honeys may contribute trace amounts of this essential nutrient and show potential health benefits. Choline plays a key role in cell membrane integrity, lipid metabolism, and signalling processes [59]. Biologically, choline acts as a precursor of the neurotransmitter acetylcholine, as a methyl donor in homocysteine metabolism, and maintains membrane integrity in muscle tissues [59]. In 2008, the USDA Choline Database reported the presence of total choline in honey at a concentration of 2.2 mg/100 g of food [60]. Another study also reported the incidence of choline in honey samples ranged between 5.94 and 36.09 mg/kg using UHPLC-MS/MS, with a limit of detection (LOD) and limit of quantification (LOQ) of 0.5 mg/kg and 1.0 mg/kg, respectively [61]. Choline was not detected in the ACH using ICS, implying that this honey was not a significant dietary source of choline. Method reliability was confirmed using a standard control with reading falls within 2SD of the mean values. The non-detection may likely indicate that choline concentration was below the quantification limits (LOD = 1.65 mg/kg; LOQ = 4.99 mg/kg) rather than a definite absence. Meat, poultry, fish, and dairy products are the primary sources of choline, with smaller amounts coming from plants such as soybeans and potatoes [58]. Honey’s potential as a source of choline may be determined by quantifying this essential nutrient.

### 4.2. Enzyme Analysis

The inhibition of certain specific digestive enzymes is of utmost importance in choosing anti-obesity and anti-diabetic agents. Orlistat and acarbose, used as a control, are two common commercial drugs used to manage obese and diabetic patients. They work by inhibiting digestive enzymes such as pancreatic lipase, α-amylase, and α-glucosidase activities in the small intestine [62].

The concentration of 0.063 mg/mL of honey was the optimum concentration needed to produce the highest inhibition (43.4%) of pancreatic lipase at concentrations ranging from 0.004 to 0.5 mg/mL. A much higher concentration of honey (e.g., 0.5 mg/mL) was found ineffective in reducing the lipase activity, due to several unknown reasons. It might not be owing to the activation of the enzyme. Still, it could be due to a certain unspecified interaction between specific bioactive components in the honey and the enzyme. The unexpected increase in lipase activity detected at higher honey concentration may be due to decreased turbidity or precipitation at higher concentrations, potentially affecting spectrophotometric readings, leading to a false interpretation of enzyme activity.

As with pancreatic lipase, inhibition of α-amylase activity also shows a similar trend. Although not substantial, higher enzyme activities were observed at 8.0 mg/mL. This could be due to some synergetic interactions happening in the solution, which are still unknown.

The ACH inhibited the α-glucosidase in a dose–response manner, although not as efficiently as the positive control acarbose. The components or substances that cause enzyme inhibition act more effectively or efficiently at higher concentrations, with more inhibitors acting upon the α-glucosidase active sites [63]. Acarbose, on the other hand, acts competitively towards the α-glucosidase active sites, which are in the same location as the substrates (carbohydrates). Due to acarbose’s high affinity for the enzyme active site, it binds more efficiently to the enzyme and prohibits the enzymatic reaction of hydrolysing carbohydrates to simple sugars that can be absorbed through the blood vessels [64].

The inhibition of α-glucosidase activity by the undiluted (unextracted) ACH and deionised water-extracted ACH at various temperatures did not show any discernible impact on the inhibitory potential. All samples exhibited a comparable inhibitory percentage, suggesting that the process of extracting bioactive compounds from the samples at specific temperatures of the extracting solution was not required to obtain a more effective impact on α-glucosidase. One possible explanation is that the bioactive compounds in the honey were readily available even without an extraction process, enabling them to be released into the solution and inhibit the activity of α-glucosidase.

Although not as efficient or significant as orlistat and acarbose, ACH effectively inhibited pancreatic lipase, α-amylase, and α-glucosidase within a specific concentration range, providing optimism for its potential use as an anti-obesity and anti-diabetic agent in the future. Since orlistat and acarbose are single-compound medications that are effective against pancreatic lipase, α-amylase, and α-glucosidase, the reduced efficacy in comparison to the positive control was anticipated. In contrast, honey is a complex blend of diverse substances that may each have unique abilities and mechanisms for enzyme inhibition. The concentrations tested (0.004–100 mg/mL) are within the range commonly used to assess antioxidant and enzyme inhibitory activities of honey and phenolic extracts, which aligns with earlier research on *A. mellifera* [65,66] and stingless bee honey [67]. *A. cerana* honey, which has the highest phenolic content among local bee species of the same region, exhibits greater α-glucosidase inhibition [68], contains polyphenols (phenolic acids, abscisic acid, and flavonoids) that contribute to its inhibitory activity [69,70,71]. However, the physiological significance remains unknown. These concentrations are primarily indicative of potential bioactivity rather than direct therapeutic implications or in vivo efficacy. This is due to differences in absorption and metabolism, where the complex gastrointestinal environment differs from in vitro conditions. These findings indicate that ACH contains bioactive compounds with potential mechanisms of action; however, they do not demonstrate therapeutic efficacy. To determine whether ACH has meaningful or significant anti-obesity or anti-diabetic effects, further research, including pharmacokinetic and pharmacological studies, in vivo experiments under physiological conditions, and potentially human clinical trials, is necessary.

## 5. Conclusions

This study has some limitations due to the small sample size. The sample, which comes from a specific location, may not reflect the natural diversity found in honey from different sources (e.g., geography, floral origin, or seasonal variations) and, therefore, cannot be generalised to all honey. However, this preliminary study can provide useful baseline data on *Apis cerana* honey (ACH) as a starting point for further research. This ACH from a tropical region in Malaysia meets the requirements for several parameters, including the sum of fructose and glucose, sucrose, and electrical conductivity as dictated by national (Malaysian Food Act) and international (Codex Alimentarius) standards. However, the moisture, free acidity, and total ash readings were higher than the recommended limits, possibly due to the climate, bee species, and botanical origin of the honey, suggesting traits more typical of honeydew honey. ACH showed considerable inhibitory activity against pancreatic lipase, α-amylase, and α-glucosidase in vitro, particularly at certain concentrations. These findings suggest potential use of ACH as a natural agent for managing obesity and type 2 diabetes. Notably, extraction with deionised water at different temperatures had no significant effect on α-glucosidase inhibition, indicating the existence of thermally stable bioactive compounds. While the results are encouraging, further in vivo studies are necessary to confirm these effects under physiological conditions. Future research should focus on improving extraction methods, identifying the specific bioactive compounds responsible for enzyme inhibition, mechanisms of action, and establishing optimal doses for potential therapeutic use.

## Figures and Tables

**Table 1:** Physicochemical analysis of ACH and choline content compared with the national and international standards for honey.

Analysis	Result (ACH)	Malaysian Food Act 1983 (Act 281) & Regulations (as of 15 August 2013) [28]	Codex Alimentarius 2009 (CXS 12-1981) [20]
Moisture (Refractometer)	***22.7 ± 0.1%***	<20%	<20%
			<23% (Hea)
Moisture (Air-oven drying)	24.4 ± 0.2%	NA	NA
Moisture (Moisture analyser)	8.6 ± 0.5%	NA	NA
Baume (°Be′)	40.1 ± 0.0 °Be′	NA	NA
Brix concentration (Brix%)	75.5 ± 0.1%	NA	NA
Fructose (F)	29.75 ± 0.27 g/100 g	NA	NA
Glucose (G)	28.76 ± 0.23 g/100 g	NA	NA
Sum of F and G	58.81 ± 0.50 g/100 g	≥60%	≥45 g/100 g (H, H/B)
			≥60 g/100 g (B)
F/G ratio	1.03	NA	NA
Sucrose	8.44 ± 0.06 g/100 g	<10%	<10 g/100 g (refer to content)
			<5 g/100 g (B)
			<15 g/100 g (L, Bor)
Maltose	0.59 ± 0.00 g/100 g	NA	NA
Lactose	ND	NA	NA
pH	4.13 ± 0.01	NA	
Free acidity	***93.7 ± 0.6 meq/1000 g***	<40 meq/1000 g	<50 meq/1000 g
Ash	***2.8 ± 0.5 g/100 g***	<1%	<1.0 g/100 g
Electrical conductivity	1.66 ± 0.00 mS/cm	NA	≥0.8 mS/cm (H, C, H/C)
			<0.8 mS/cm (B)
Colour analysis	150.0 ± 0.0 mm Pfund (Dark Amber)	NA	NA
Choline	ND	NA	NA

Sum of F and G—Sum of Fructose and Glucose; F/G ratio—Fructose/Glucose ratio; meq/1000 g—milliequivalents acid/1000 g; NA—Not available; ND—Not detected; below the detection limit of 1.65 mg/kg; B—Blossom honey; H—Honeydew honey; H/B—Blend of Honey; Hea—Heather honey; L—Lavender honey; C—Chestnut honey; Bor—Borage honey. The bold *italic* features represent the exceeding values from the honey standards.

## Data Availability

The data presented in this study are available on request from the corresponding author due to privacy.

## Abbreviations

The following abbreviations are used in this manuscript:

ACH	*Apis cerana* honey
IMR	Institute for Medical Research
NIH	National Institutes of Health
HPLC	High-performance Liquid Chromatography
IHC	International Honey Commission
USDA	United States Department of Agriculture
ICS	Ion Chromatography System
SPB	Sorenson’s Phosphate Buffer
PNPG	4-nitrophenyl β-D-glucopyranoside
MFA	Malaysian Food Act 1983
CA	Codex Alimentarius 2009
F	Fructose
G	Glucose
F/G	Fructose and Glucose ratio
